# Comparison of Insertional RNA Editing in *Myxomycetes*

**DOI:** 10.1371/journal.pcbi.1002400

**Published:** 2012-02-23

**Authors:** Cai Chen, David Frankhouser, Ralf Bundschuh

**Affiliations:** 1 Biophysics Graduate Program, The Ohio State University, Columbus, Ohio, United States of America; 2 Department of Physics, The Ohio State University, Columbus, Ohio, United States of America; 3 Department of Biochemistry, The Ohio State University, Columbus, Ohio, United States of America; 4 Center for RNA Biology, The Ohio State University, Columbus, Ohio, United States of America; Ottawa University, Canada

## Abstract

RNA editing describes the process in which individual or short stretches of nucleotides in a messenger or structural RNA are inserted, deleted, or substituted. A high level of RNA editing has been observed in the mitochondrial genome of *Physarum polycephalum*. The most frequent editing type in *Physarum* is the insertion of individual Cs. RNA editing is extremely accurate in *Physarum*; however, little is known about its mechanism. Here, we demonstrate how analyzing two organisms from the *Myxomycetes*, namely *Physarum polycephalum* and *Didymium iridis*, allows us to test hypotheses about the editing mechanism that can not be tested from a single organism alone. First, we show that using the recently determined full transcriptome information of *Physarum* dramatically improves the accuracy of computational editing site prediction in *Didymium*. We use this approach to predict genes in the mitochondrial genome of *Didymium* and identify six new edited genes as well as one new gene that appears unedited. Next we investigate sequence conservation in the vicinity of editing sites between the two organisms in order to identify sites that harbor the information for the location of editing sites based on increased conservation. Our results imply that the information contained within only nine or ten nucleotides on either side of the editing site (a distance previously suggested through experiments) is not enough to locate the editing sites. Finally, we show that the codon position bias in C insertional RNA editing of these two organisms is correlated with the selection pressure on the respective genes thereby directly testing an evolutionary theory on the origin of this codon bias. Beyond revealing interesting properties of insertional RNA editing in *Myxomycetes*, our work suggests possible approaches to be used when finding sequence motifs for any biological process fails.

## Author Summary

RNA is an important biomolecule that is deeply involved in all aspects of molecular biology, such as protein production, gene regulation, and viral replication. However, many significant aspects such as the mechanism of RNA editing are not well understood. RNA editing is the process in which an organism's RNA is modified through the insertion, deletion, or substitution of single or short stretches of nucleotides. The slime mold *Physarum polycephalum* is a model organism for the study of RNA editing; however, hardly anything is known about its editing machinery. We show that the combination of two organisms (*Physarum polycephalum* and *Didymium iridis*) can provide a better understanding of insertional RNA editing than one organism alone. We predict several new edited genes in *Didymium*. By comparing the sequences of the two organisms in the vicinity of the editing sites we establish minimal requirements for the location of the information by which these editing sites are recognized. Lastly, we directly verify a theory for one of the most striking features of the editing sites, namely their codon bias.

## Introduction

RNA editing describes the process in which individual or short stretches of nucleotides in a messenger or structural RNA are inserted, deleted, or substituted. As a consequence, the final RNA product translated into a protein or functional by itself is different from its genomic template in organisms with RNA editing. RNA editing is widely spread across species, including plants, mammals, slime molds, viruses and many other organisms [1]–[7]. In some organisms, RNA editing is essential for their survival while for others it provides another layer of fine tuning the genetic program. Although some distinct editing mechanisms have been identified, in many instances the mechanisms of RNA editing are not understood at all [2], [5], [7].

A high level of RNA editing has been observed in the mitochondrion of the slime mold *Physarum polycephalum* [1], [4], [8]–[10]. In this organism, the mRNA of nearly every mitochondrial protein coding gene is edited at a rate of approximately one out of 25 nucleotides, while structural RNAs are edited at a rate of on average one out of every 40 nucleotides [1], [4], [8]. The by far most frequent editing type in *Physarum* is the insertion of individual Cs. However, the mitochondrion of *Physarum* performs a whole set of other editing types, including insertion of individual Us, insertion of certain dinucleotide pairs, deletion of nucleotides and substitutions of Cs by Us [10]–[12]. It has been shown *in vivo* that RNA editing in *Physarum* is extremely accurate [13], i.e., that nearly every transcript is completely edited at exactly the correct position.

While the machinery inside the mitochondrion of *Physarum* recognizes the editing sites with extreme precision, we know neither the mechanism by which these editing sites are recognized nor what machinery is actually performing the editing. It is challenging to decipher the code that determines the editing sites and identify the machinery that performs the actual editing. As far as the machinery is concerned, it has been determined that editing in *Physarum* is co-transcriptional, i.e., that the RNAs are edited as they are synthesized [14], [15]. Thus, the RNA editing machinery should be part of the RNA polymerase itself or very closely associated with it. As far as the location of the editing sites is concerned, it has been determined that the recognition of editing sites and the actual editing are two independent processes [16]. In order to understand the RNA editing machinery, it is necessary to identify how the RNA editing machinery knows which sites to edit. It is known that only the DNA in close proximity of the site is necessary in order to obtain editing [17]. Rhee *et al.* [18] demonstrated that DNA necessary for C insertion is within 9 or maybe 10 base pairs on either side of the editing site. But no sequence patterns have been identified that could explain how the sites are recognized. Although no patterns have yet been identified, computational methods for the prediction of insertional editing sites have been developed [19]–[21]. These prediction methods do not require any knowledge about the mRNA. They have been shown to predict the protein sequence with an accuracy of as much as 90% [19].

Here we present the comparison of insertional RNA editing in two related organism in *Myxomycetes*: *Physarum polycephalum* and *Didymium iridis*. Sequence information on mitochondrial genes of *Didymium iridis* has recently become available [22]–[24] and can be compared to the also recently determined complete edited transcriptome of *Physarum polycephalum* [25]. Being able to compare sequences from two related organisms promises better understanding of the editing machinery that is vital to the successful function of these organisms.

Our contributions include the prediction of RNA editing sites in *Didymium* genes based on the knowledge of the *Physarum* mitochondrial transcriptome, the investigation of sequence conservation in the vicinity of editing sites, and an analysis of codon bias. We use a computational approach to “predict” RNA editing sites in 15 *Didymium* genes for which the editing sites are known and find that using the *Physarum* protein sequence can greatly increase the prediction accuracy of *Didymium* editing sites. We also predict 7 new *Didymium* genes and their editing sites, and the prediction results suggest one of these genes may be unedited. We investigate the sequence conservation in the vicinity of editing sites between two organisms. Our data implies that a local RNA editing recognition mechanism that is based only on the information contained in any combination of the 18 nucleotides in immediate vicinity of an insertional editing site, even one that uses a different recognition agent (such as a guide RNA or a protein) for every single site, is unlikely. In addition, we show that if such a mechanism exists it has to use nearly all of the 18 positions to specify the site. Finally, we examine the codon position bias in C insertional RNA editing of these two organisms. A strong relationship between the strength of the codon bias and the overall sequence conservation is reported: more conserved genes tend to have more significant codon bias. This result verifies a previous mutation-selection theory for the codon bias.

The recognition mechanism of insertional RNA editing in *Myxomycetes* is an example where searches for common sequence motifs in a single organism have failed in spite of a very large number of training sites leading to the conclusion that site specific recognition mechanisms must be at work. This situation is not specific to the case of insertional RNA editing but can occur generically in any search for biological sequence motifs. Thus, the work presented here is not only interesting in terms of the specific results on insertional RNA editing in *Myxomycetes* but also much more broadly in terms of strategies to be employed if biological sequence motif searches in individual organisms fail.

## Results/Discussion

### RNA editing site prediction accuracy in *Didymium*

The first issue we address is to what extent the recently achieved complete knowledge of the edited *Physarum* transcriptome [25] improves computational prediction of genes and editing sites in the *Didymium* mitochondrial genome. Computational prediction of insertional RNA editing in the *absence* of a reference transcriptome has been presented before [11], [19], [20]. The method uses a position specific scoring matrix (PSSM) built from protein sequences from other organisms. It finds the editing sites for a given genomic sequence that translate to the putative protein with maximum similarity to the protein family described by the PSSM. With this method, we prepared predictions of editing sites in *Didymium* using the PSSMs made before the *Physarum* transcriptome was known [11], [19], [21]. Since the edited *Physarum* transcriptome is now known [25], new predictions utilizing that information were created and compared to these baseline PSSM predictions. Given that *Physarum* and *Didymium* both exhibit RNA editing and are closely related, the purpose of this process was to see how much the new predictions, which include transcriptome information from *Physarum*, improve when compared to the baseline.

In order to be able to evaluate and compare the prediction quality we applied our prediction methods to mitochondrial genes in *Didymium* for which the editing sites had already been determined experimentally [22], [23]. In total we created five predictions of editing sites for every gene. The first was the baseline prediction using the PSSM developed before the *Physarum* transcriptome was known as described above. The second was a *Physarum* based PSSM. The *Physarum* based PSSMs were created from the NCBI website by starting a PSI-BLAST search [26] with the homologous *Physarum* protein sequence for each gene rather than with a homologous protein sequence from a more distant organism as in the creation of the original PSSMs. Three iterations of a protein PSI-BLAST search were run for each of the sixteen genes for which the *Didymium* editing sites are known (see Methods section). During this process we manually excluded the *Didymium* protein for which the prediction was being made from the model building. Since the first round of PSI-BLAST did not find any homologs when starting from the *Physarum* protein sequence for atp8, we could not create a *Physarum* based PSSM for atp8 and excluded it from further analysis. The remaining three predictions did not use a PSSM summarizing the properties of a whole family of homologs. Instead, the plausibility of a putative *Didymium* protein sequence (generated by inserting Cs into the *Didymium* genomic sequence and translating the result) was quantified by aligning the putative *Didymium* protein directly to the known *Physarum* homolog. Since alignment scores depend on the scoring matrix and different matrices are tuned toward different evolutionary distances, we prepared one prediction each using the BLOSUM62, BLOSUM75, and BLOSUM90 matrices.

**Figure 1:**
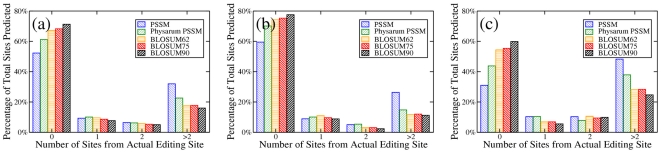
Accuracy of different prediction methods of insertional RNA editing sites in *Didymium*. Each graph shows the percentage of editing sites which are correctly predicted, predicted by one, two, or at least three positions away from the experimentally known correct editing site. (a) shows results for all 15 genes studied, (b) for the more conserved genes, and (c) for the less conserved genes.

We scored the accuracy of a prediction by counting the number of correct and incorrect predictions made by each prediction method (see Methods). We report the results as a percentage of editing sites in each category relative to the number of predictions the method included overall. The percentage of predicted editing sites is a better indicator than the absolute number since the computational model often does not make predictions for editing sites near the ends of genes. The results for each of the five prediction methods among all fifteen considered genes are shown in Figure 1(a).

Figure 1(a) indicates that the PSSMs created without using *Physarum* generate the worst predictions showing that the inclusion of the *Physarum* genome does increase the accuracy of the prediction; a finding that is expected due to the similarities between the organisms and their shared RNA editing. Interestingly, the results show that predictions using the *Physarum* protein alone outperform the predictions using either of the two PSSMs. Among the predictions that use only the *Physarum* protein the prediction accuracy increases as the BLOSUM matrices are tuned toward more closely related organisms. This result implies that the organisms are so similar that the inclusion of the genetic information of other organisms into the PSSMs actually decreases the accuracy of the editing site predictions.

Since the prediction method relies on sequence homology we wanted to determine the influence of sequence similarity on the prediction quality. To this end we separated the fifteen genes into a more conserved and a less conserved group (see Table S1) based on the nucleic acid conservation of the second codon position between the known *Physarum* and *Didymium* mRNA sequences. The prediction accuracy for all five methods in each of these two groups is shown in Figure 1(b) and (c).

The overall trend in prediction accuracy is the same for the more highly conserved and the more diverged set of genes. However, although one might have expected that using the *Physarum* protein works better for genes where the two organisms have diverged less from each other, the improvement in prediction accuracy by using the *Physarum* protein sequences is actually bigger for the more diverged set of genes than for the more conserved set of genes. We rationalize this by arguing that genes with less conservation between *Physarum* and *Didymium* are generally under less evolutionary pressure and thus will also have diverged more between the *Myxomycetes* in general and the other organisms used to build the baseline PSSMs. Thus, including proteins from other organisms in the prediction hurts the prediction accuracy more for less conserved genes.

### Prediction of new *Didymium* genes

**Figure 2:**
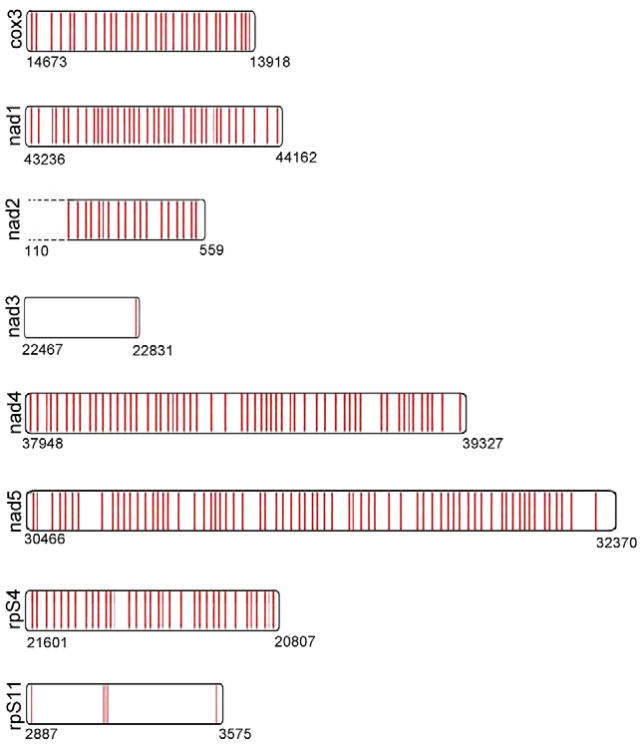
Graphical representation of the positions of predicted editing sites. These predicted editing sites are in the seven newly identified *Didymium* mitochondrial genes as well as in nad3 for which it is experimentally known that it is unedited in *Didymium* [24]. The predictions for the nad2 gene are incomplete due to a lack of genomic sequence, indicated by the dashed lines for that gene.

Encouraged by the quality of the predictions on the already known *Didymium* genes, we proceeded to use the method to search for other genes in the *Didymium* mitochondrial genome and to predict the editing sites that are present in those genes. In order to do this, the genes first had to be located within the partial *Didymium* mitochondrial genome available to us. This was accomplished by scoring segments of the partial genome against the corresponding proteins from *Physarum* as described before [20]. We used only the *Physarum* sequence and the BLOSUM90 matrix since this approach performed best on the known genes as described above. Once the location of the genes were identified as the segments with the highest score, the editing sites were predicted. The results are shown in graphical form for each of the eight genes we identified in Figure 2. The predicted mRNA sequences with C insertions indicated as upper case C's are given in Table S2. We note that the predictions for nad2 only include part of the gene; a region at the 5′ end is not present as it is missing from the partial genomic DNA sequence available to us.

The two genes that stand out by their very low number of editing sites are nad3 and rpS11. Indeed, nad3 is already known to be unedited [24]. Our prediction resulted in a single editing site in nad3 toward the end of the gene. While this addition of a single predicted editing site was not expected, it is understandable since the prediction of editing sites becomes more challenging toward the ends of the gene. Six editing sites were found in rpS11; one was found near the 5′ end, three in close proximity of each other in the middle, and two were at the 3′ end. Because of the low number of editing sites and the striking pattern of the predicted editing sites we hypothesize that rpS11 is also unedited just like nad3. The additional predicted editing sites at the end are easily understood based on the overall low prediction accuracy at the end of genes. Since the three predicted editing sites in the middle of the gene are close to each other they can also be a prediction artifact; omitting them would only change the protein sequence over the range of 5 amino acids. We verified that omitting the three editing sites would not create an in frame stop codon in the middle of the protein. Thus, is is plausible that the edited rpS11 mRNA could have been reverse transcribed and inserted into the genome of *Didymium* as it has been hypothesized for nad3 [24]. We note, however, that while nad3 also has a very much reduced number of editing sites in *Physarum* (around one every 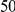 nucleotides rather than the usual one every 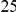 nucleotides), rpS11 shows the normal level of editing in *Physarum*.

While the graphs presented in the preceeding section convey the successes of the various computational methods for predicting the editing sites in the genes of *Didymium* with known editing sites, there can be no similar comparison of the successes of the predictions in the new genes shown here as their exact editing sites are not known. However, sequence alignment of the new genes does show that of these genes, rpS4 and rpS11 fall into the less conserved group while cox3, nad1, nad2, nad4, and nad5 (as well as nad3) all fall into the more conserved category. Using this information, an estimate about the success of the BLOSUM90 computational method can be made for the new genes based the known results of the BLOSUM90 prediction method for the sequenced genes. This estimate results in Figure S1 which show the expected number of correct sites, the expected number of sites one or two sites from the actual editing site, and the expected number of more wrongly predicted sites with the associated errors. A true assessment of the success of these predictions will of course have to wait until these RNAs are fully sequenced and their editing sites are known.

### Sequence conservation in the vicinity of editing sites

As indicated in the introduction, one of the major questions to be resolved is how the RNA editing machinery knows which sites to edit. Previous studies [10], [11], [25] have looked for sequence patterns in *Physarum* alone. One property within the mitochondrial genome of *Physarum* is that editing sites have a strong preference to occur after a combination of a purine and a pyrimidine [10], [11]. However, many editing sites do not follow this pattern and many purine-pyrimidines are not followed by an editing site. Thus, this pattern alone cannot explain the extremely reliable recognition of editing sites and the problem of editing site recognition remains unsolved.

The absence of discernable sequence patterns among the *Physarum* editing sites might suggest that every site (or small groups of them) are recognized individually. Such mechanisms exist in the kinetoplastids in the form of guide RNAs [27], [28] and in plant chloroplasts and mitochondria in the form of PPR proteins [6], [7], [29]. If every editing site is recognized individually, no sequence pattern will emerge when comparing the sequences surrounding all the editing sites in one organism consistent with previous studies in *Physarum* [10], [11], [25]. However, when comparing *organisms* at sequences surrounding their *shared* editing sites, sequence positions that play a role in site recognition are under increased evolutionary pressure and should thus show more conservation across species than sequence positions not involved in editing site recognition. Thus, instead of looking at one organism at a time, we here used the edited genes of two related organisms with insertional RNA editing, *Physarum* and *Didymium*, and examined the patterns of sequence *conservation* between the two organisms. We looked at the nucleotide identities at fixed positions relative to the editing site and investigated whether these nucleotides were conserved between the two organisms or not. In this analysis, we tried to identify positions relative to the editing site with statistically significantly increased degree of conservation from the background, which would indicate functional importance.

We studied the sixteen genes for which the editing sites are known in both organisms as described in the Methods section. Comparing the complete mRNA sequences of the two organisms, we determined the overall degree of conservation for the first, second and third codon position. This yielded the background frequencies or the “expected” frequencies at the first, second, and third codon position. Table 1 presents these background frequencies for conservation between *Physarum* and *Didymium*. The degree of conservation in these genes is relatively high and may not leave enough room to be significantly increased. Thus, we also studied as another group the subset of the 8 less conserved among the 16 genes (i.e., the genes the background frequency at the 2nd codon position of which is less than 85%, see the Methods section). It can be seen in table 1 that the two groups share similarities in their background levels of conservation that are to be expected: the second codon position has the highest conservation, while the third codon position is the least conserved. However, there is a clear difference in the amount of conservation between the two groups as expected by construction of the less conserved group.

**Table 1:** Background frequencies for conservation between *Physarum* and *Didymium*.

Codon position	“all” genes	“less conserved” genes
first	76.9%	68.0%
second	84.8%	74.3%
third	65.1%	63.3%

In order to study the vicinity of the editing sites shared by the two organisms, we first identified those C insertional editing sites that are shared and unambiguous (i.e., at least in one of the two organisms the neighboring nucleotides are not Cs). Because of the variations of background frequencies among different codon positions, these editing sites were separated by codon position. Table 2 shows the number of these shared editing sites for each codon position.

**Table 2:** Number of C insertional editing sites shared by *Physarum* and *Didymium*.

Codon position	“all” genes	“less conserved” genes
first	90(34.1%)	45(38.8%)
second	26(9.8%)	17(14.7%)
third	148(56.1%)	54(46.6%)

A previous study demonstrated that the DNA necessary for C insertion is contained within 9 or maybe 10 base pairs on either side of the editing site [18]. Thus, we first determined the conservation information of the flanking sequences within a window of 9 positions upstream and downstream of each of the shared editing sites. Then we examined the difference between the observed sequence conservation in the vicinity of the shared insertional editing sites (at positions −9 to +9 relative to editing sites) and the background conservation.

**Figure 3:**
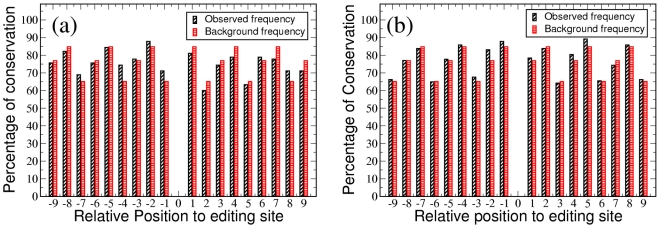
Comparison of observed and expected conservation. The observed conservation and background conservation for all 16 genes are compared for editing site at the (a) first and (b) third codon position.

Figure 3 shows the observed and the expected degree of conservation for positions −9 to +9 (relative to the shared editing site) for all genes separately for the shared editing sites at the first and third codon position (we do not show the data for the second codon position because of the small number of these editing sites which results in very low statistical significance). Results for the less conserved group are similar to the results for all genes (see Figure S2). From these figures, we can see that both the observed frequency and the background frequency are position dependent with codon position being the dominant factor. The observed frequency is higher than the background frequency at some positions, while at other positions the observed frequency is lower.

**Figure 4:**
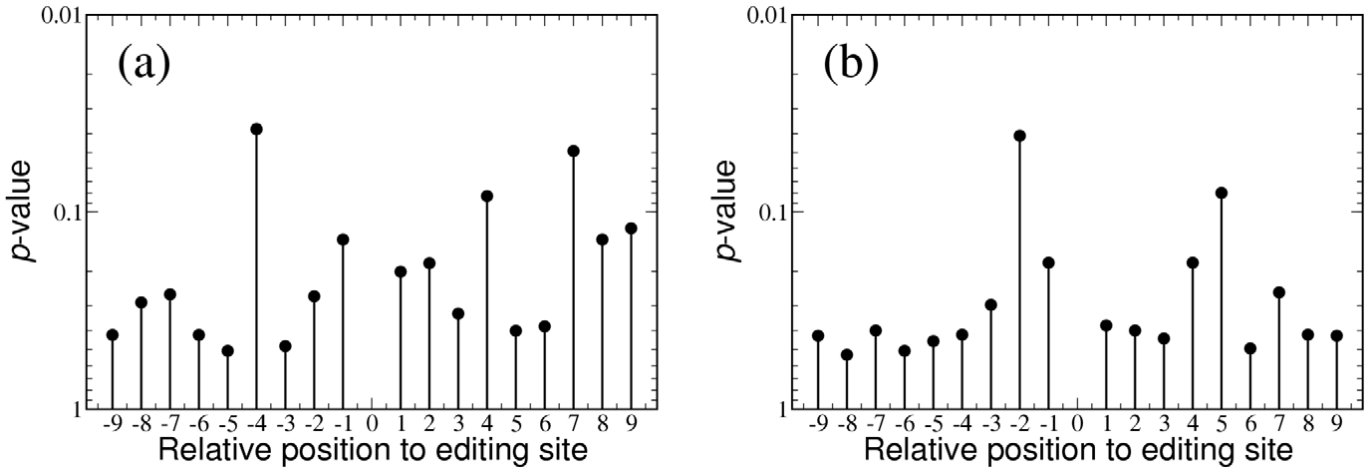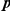-values for the differences between the observed and the background conservation. These 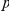-values are calculated for shared editing sites in all 16 genes at the (a) first and (b) third codon position. The threshold for statistical significance (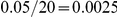 as the 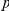-value cut off) is not indicated in the figure as it is far above the top of the graphs.

In order to see whether these variations are statistically significant, we calculated the probabilities for observing increased or decreased sequence conservation in the vicinity of the shared insertional editing sites based on the binomial distribution (see details in the Methods section). Figure 4 shows those probabilities for positions −9 to +9 for all genes. No significant 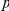-values are obtained (to take into account multiple testing, we use 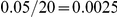 as the 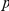-value cut off), which implies that there are no statistically significant variations between the observed frequencies and the background frequencies. In spite of the larger room for increased conservation, the results for the less conserved group are similar to the results for all genes (see Figure S3).

We also extended our study to positions that are further away from the editing site than 9 nucleotides. For these positions, the analysis is complicated by the fact that additional editing sites can occur between the position to be studied and the editing site of interest thereby mixing different codon positions at the same position relative to the editing site of interest [10]. We circumvent this problem by eliminating all primary editing sites from the analysis that have an additional editing site between the primary site and the position we are interested in. The disadvantage of this approach is that as one studies positions that are further and further away from the primary editing site, there are less and less sequences that contribute to the analysis and thus the statistical power decreases. In practice, we reached a limit of 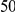 contributing sequences at a distance of 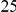 nucleotides for editing sites at the third codon position and at a distance of 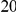 nucleotides for editing sites at the first codon position. However, even for these distances no statistically significant increase of sequence conservation was found (data not shown). This suggests that the information on editing site location is not contained within the sequence in the immediate vicinity of the editing site at least at the level of statistical significance set by our sample size.

This leaves us with the conundrum that on the one hand Rhee *et al.* [18] demonstrate experimentally that only 9 nucleotides of DNA on either side of the editing site are required for editing and on the other hand our results suggest that there is no statistically significant pattern of conservation within 9 (or even more) base pairs on either side of the editing site. Thus, we propose several possible explanations for the discrepancy between Rhee *et al.*'s findings and our results.

1. Given that we only analyze 16 genes, our sample size might not be large enough to obtain results with high level of statistical significance.
2. The RNA editing machinery does not recognize the same positions relative to the editing site at all of the sites. In this case, the increase in conservation in any fixed position may be too small to observe.
3. The templates for editing site recognition in the two organisms are their own mRNA molecules. In this case, there is no need for increased conservation in the vicinity of the editing sites (or anywhere else). However, it is hard to envision an actual mechanism that uses the mRNA molecules themselves and that is compatible with the known co-transcriptional editing in *Physarum* [14], [15].
4. Editing site recognition contains two steps: First, the RNA editing machinery recognizes the recognition sites for editing that are located far away from editing sites (at least outside the base pair range studied here) *before* the gene is transcribed, and the machinery will then put a marker (used for directing RNA polymerase) into the region within 9 base pairs of editing sites. At the time of transcription, the marker will then direct RNA polymerase to initiate the editing event. In this case, the editing *information* is located far away from the editing sites. But once the marker is set, it is the *DNA* within 9 bases of the editing sites that carries the marker and thus directs the editing as observed by Rhee *et al.* We call this hypothesis the “marker model”.

The first and second explanation lead us to consider how much increase in conservation for recognition sites of editing events we should see given the size of the mitochondrial genome of *Physarum* (62862 bp). Due to the extreme precision of RNA editing in *Physarum*, the recognition site of each editing event should be unique in the mitochondrial genome of *Physarum*. According to Rhee *et al.*, the 9 nucleotides immediately upstream DNA and 9–10 nucleotides immediately downstream DNA of the editing sites are necessary and sufficient for editing site recognition. If the actual *information* on the editing site position is stored within these nucleotides this implies that the pattern recognized within the set of 18–19 nucleotides should occur at the rate of at most 1/60000 in a random DNA sequence.

Based on our calculations (see Methods section), the lowest conservation for a set of 19 nucleotides that still allows specification of a site within the genome is 80.7%, i.e., at least we should see 80.7% conservation in a 19 nucleotide region responsible for editing site recognition. To test whether a conservation of 80.7% or more would show up as a significant difference between the expected frequencies and the background frequencies at our sample size, we set 80.7% (the lowest expected conservation) as the “observed frequency” for positions −9 to +9 (i.e., we used 80.7% to replace the real observed frequencies). Then we calculated the 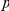-values for observing increased sequence conservation relative to the background frequencies in Table 1. For putative motif positions at the third codon position in the vicinity of editing sites at the third codon position we found a highly significant (compared to the cutoff of 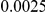) 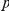-value of 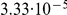. Thus, according to this analysis, we should have seen statistically significant variations between the actual observed frequencies and the background frequencies at our sample size even if the editing machinery does not recognize the same positions relative to the editing site at all of the sites. We thus conclude that the observed degree of conservation is significantly lower than what is to be expected when only the 9 nucleotides upstream and 10 nucleotides downstream of the editing sites contain the information for editing site recognition even if different sites use different combinations of the 19 nucleotides to specify the editing site location. These studies therefore suggest that the first and second of the hypotheses above can be ruled out.

As another test of which aspect of sequences around editing sites could determine the editing position, we tested the specificity of sequences around editing sites. In practice, we started by looking only at sequences immediately downstream of editing sites and examined the uniqueness of these sequences in *Physarum*, that is, we tested for every sequence of 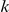 nucleotides (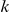-mer) downstream of an unambiguous C insertion site in the known transcriptome of *Physarum* if this 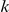-mer only occurs downstream of C insertional editing sites, but does not occur following non-edited sites of the sequences. This analysis is especially powerful since the full transcriptome has recently been determined by a high throughput sequencing experiment [25] thereby giving complete access to all editing and non-editing sites for this analysis that compares all editing sites to all non-editing sites in all transcripts.

Since it is unknown if editing site recognition occurs at the DNA or RNA level, we tested the 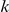-mers in both the unedited sequences and the edited sequences. We asked which is the largest 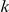 for which we can still find a 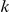-mer that occurs at least once immediately downstream of an unambiguous C insertion site and at least once in a position that is definitely not preceded by an editing site. Both on the RNA and on the DNA level the largest 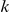-mer we found was a 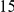-mer. Thus, we conclude that a mechanism that uses only the downstream sequence of an editing site to specify the editing event, even if it is a different mechanism for every editing site, must use at least 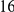 nucleotides downstream of the editing site. Similarly, we found one 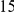-mer combination that is not unique for unambiguous C insertional editing sites when testing the unedited sequences and one 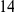-mer when testing the edited sequences when studying only sequences immediately upstream of the unambiguous editing sites.

Given that Rhee *et al.* found that the 9 or maybe 10 nucleotides of DNA both downstream and upstream of the editing sites are responsible for the editing event, we also investigated the specificity of all the possible nucleotide combinations upstream and downstream of the unambiguous C insertional editing sites within 9 nucleotides on either side (describing them as 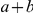, where 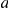 is the number of nucleotides upstream of the editing site and 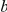 is the number of nucleotides downstream of the editing site with 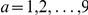 and 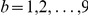). Since in this case the unambiguous C is inserted inside the motif, testing the uniqueness of these motifs is different between the unedited sequences and the edited sequences. It is the same as before for the unedited sequences. For the edited sequences, we asked if the motif *without* the inserted C occurs anywhere in the transcriptome (in addition to the at least one occurence *with* the inserted C). We found possible combinations that are not unique for the unambiguous C insertional editing sites for both unedited sequences and edited sequences up to 9+5, 8+7, 7+8 and 6+9 nucleotide combinations. Therefore, the recognition region (if it exists) includes at least 9+6, 8+8, or 7+9 positions in agreement with Rhee *et al.*'s finding [18] that the whole 9+9 nucleotides are required for editing. It is important to note that since this particular analysis does not rely on the comparison of two organisms but uses only *Physarum* data it even in the case of hypothesis 3 (that the mRNA itself templates the editing sites) implies that the recognition has to involve at least the 9+6, 8+8, or 7+9 nucleotides surrounding an editing site. We would like to conclude by noting that while we did not find a non unique 9+9-mer which would rule out the “9+9” (or any larger) model (the identity of the 9 nucleotides both downstream and upstream of the editing sites carry the information for the editing event), we would not have expected to find one on statistical grounds alone since the probability for an 18-mer to occur in the mitochondrion of *Physarum* is extremely small even if there is no biological reason (uniqueness of the recognition sequence) that prevents it from occurring. We want to emphasize again, that the conclusions in this section do not rely on a common mechanism that simultaneously recognizes all editing sites in one organism but apply even to mechanisms that recognize every editing site individually such as guide RNAs or site recognition proteins. Of course, all these considerations only exclude the *information* for the editing site positions to be stored within the identities of the 9+5, 8+7, 7+8, or 6+9 nucleotides surrounding the editing site - it is still possible that the DNA in the immediate vicinity of the edited site carries a “marker” that is placed based on information elsewhere in the genome.

### Codon bias

For editing sites within the coding regions, a significant codon bias is known. It has been found that in the mitochondrial genome of *Physarum*, the third codon position has the largest number of C insertional editing sites, while the second codon position has the lowest number [9], [10], [25], [30]. As shown in Table 2 the codon bias is also significant for the shared C insertional editing sites in both groups of genes.

A previous study proposed an evolutionary model which explains this codon position bias [31]. The general idea of this model is the following. During the proliferation of *Physarum*, nucleotide mutations (including substitutions, insertions, and deletions) occur at random positions in the mitochondrial DNA sequence. In the case of random deletions, the offspring can not survive because of the incorrect protein sequence since the mutated DNA sequence is out of frame. However, the editing machinery sometimes may insert back nucleotides to the positions of deletions and preserve the correct reading frame. In this case, the offspring can survive and proliferate. This idea of random creation of new editing sites is also consistent with phylogenetic data [32].

The net effect of a nucleotide deletion followed by the creation of a new insertional editing site is that the original nucleotide will be replaced by a C. The genetic code is organized such that the third codon position is the most irrelevant for the identity of the amino acid while the second codon position is the most relevant. Therefore, the third codon position is the least sensitive to nucleotide changes to C generated in editing events while the second codon position is the most. Thus, random deletions at the third codon position will have the highest survival rate and the lowest for the second codon position.

According to this model, the codon position bias in *Physarum* is mainly a consequence of random mutations with selection at the protein level [31]. This implies that genes that are under stronger selection should have a stronger codon position bias in their editing sites as well. Since for this study we have two organisms, we can directly determine the strength of selection on each gene from the sequence conservation. Thus, to test the theory proposed in [31], we examined the relationship between the strength of the codon bias and the overall sequence conservation in both *Physarum* and *Didymium*.

**Figure 5:**
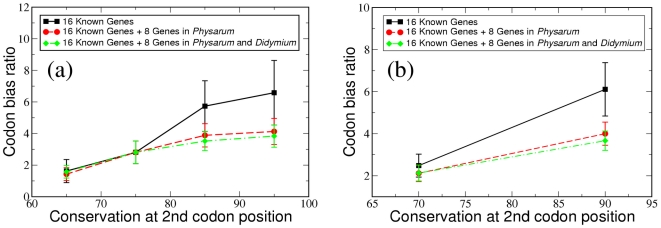
Relationship between codon bias (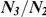) and the conservation at the second codon position. 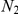
 and 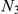 are the number of second and third codon position editing sites. Based on the conservation at the second codon position, the genes are separated into (a) four groups and (b) two groups. For the case of 16 known genes, we counted all unambiguous C insertional editing sites in *Physarum* and *Didymium*). For the case of 16 known genes + 8 genes in *Physarum*, we counted all unambiguous C insertional editing sites in *Physarum* and *Didymium* for the 16 known genes and unambiguous C insertional editing sites only in *Physarum* for the additional 8 genes. For 16 known genes + 8 genes in *Physarum* and *Didymium*, we counted all unambiguous C insertional editing sites in *Physarum* and *Didymium* for all 24 genes.

As described in the Methods section, the 16 genes were divided into several groups according to their overall sequence conservation at the second (most conserved) codon position between *Physarum* and *Didymium*. The detailed group information is shown in Table S1 (since the conservation at the second position of the 16 genes ranges from 60% to 100%, we separated them into four groups by splitting the range from 60% to 100% into four intervals of equal length). We used the ratio of the number of third codon position editing sites 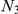 and the number of second codon position editing sites 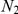 as a measure of codon bias, and used the overall sequence conservation at the second codon position as a measure of the conservation within different genes. We examined all unambiguous C insertional editing sites in *Physarum* and *Didymium*, i.e., shared editing sites as well as editing sites specific for either of the organisms. The solid black squares in Figure 5 illustrate the relationship between the codon bias 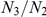 and the conservation at the second codon position. In order to reduce statistical fluctuations, we also considered a grouping of the genes into only two groups by combining data of all genes with conservation between 60% and 80% at the second codon position into one group and the genes with conservation between 80% and 100% into the other group. Figure 5 shows that, whether the 16 known genes were separated into four groups or into two groups based on their conservation at the second codon position, genes with higher conservation at the second codon position have a higher ratio of 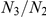. This difference is significant even when statistical errors within the ratios are taken into account. This demonstrates that genes under stronger selection (or with higher conservation) should have a stronger codon position bias in their editing sites; thus reinforcing the theory that codon bias is a consequence of evolutionary pressure on the protein sequence.

In order to increase the statistical significance it would be beneficial to include more genes in the study. Given our work presented above, the seven newly predicted *Didymium* genes and nad3 are likely candidates to add to the study. The problem with this idea is that the predicted mRNA sequences most likely deviate from the (unknown) true mRNA sequences, which might affect the accuracy for both the overall sequence conservation and the codon bias. Since we have the predicted *Didymium* mRNA sequences for all the 16 genes for which the actual mRNA sequences are known, we can test how much the estimates of overall sequence conservation and the codon bias differ between the predicted sequences and the true sequences.

**Figure 6:**
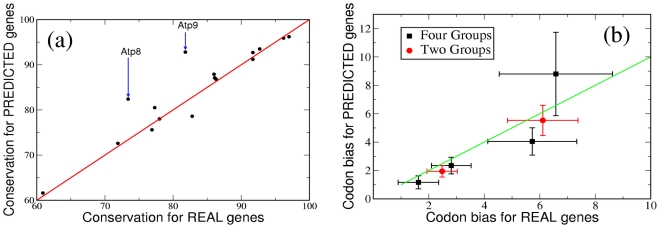
Comparison of (a) overall conservation and (b) codon bias for real and predicted mRNA sequences. The data is close to the diagonal in both cases indicating that predicted sequences can be used to estimate these quanitities in cases where the true sequences are not known.

To this end, we aligned the predicted *Didymium* mRNA sequences and the real *Physarum* mRNA sequences (see Table S3 for accession numbers) to obtain the conservation at the second codon position. Then we plotted the conservation data between the predicted *Didymium* mRNA and the real *Physarum* mRNA versus the conservation data between the real *Didymium* mRNA and the real *Physarum* mRNA. (see Figure 6(a)). We found, that the overall conservation at the second codon position for the predicted sequences (predicted *Didymium* mRNA and real *Physarum* mRNA) and the real sequences (real *Didymium* mRNA and real *Physarum* mRNA) are very close to each other except for possibly two genes – atp8 and atp9 – which are much shorter than the other genes. This implies that estimating the overall sequence conservation from the predicted *Didymium* mRNA sequences is a valid procedure since the difference in conservation by using the predicted sequences and the real sequences is small.

In the same way, we compared the codon bias between the predicted sequences and the real sequences. We treated the predicted *Didymium* sequences in the same way as the real *Didymium* sequences before (we examined all unambiguous C insertional editing sites and used four and two groups). As can be seen from Figure 6(b), the codon biases for the predicted sequences and the real sequences are equal within the error bars. This suggests that codon biases calculated from the predicted *Didymium* sequences can reasonably be used in lieu of exactly known codon biases. However, the agreement between predicted and true sequences is not as strong for the codon bias as it is for the conservation at the second codon position.

Since the overall sequence conservation and the codon bias show only small deviations between the predicted sequences and the true sequences, we can add the seven newly predicted *Didymium* genes and nad3 to the codon bias analysis. In the same way as described for the 16 known genes, we analyzed the codon bias of these eight genes using the predicted *Didymium* sequences and real *Physarum* sequences. Since the determination of conservation at the second codon position from predicted sequences is more robust with respect to prediction errors than the determination of codon bias (see Figure 6) we performed this analysis twice. First, we only used the (known) codon bias in *Physarum* for the eight predicted genes (indicated in Figure 5 as 16 known genes + 8 genes in *Physarum*), thus only using the predicted *Didymium* mRNA sequences to determine the overall conservation for group division of each gene, but not using the codon bias in the predicted *Didymium* mRNA sequences which is less robust with respect to prediction errors than that for overall conservation. Second, we also included the predicted editing sites in *Didymium* for the eight additional genes in the analysis (indicated in Figure 5 as 16 known genes + 8 genes in *Physarum* and *Didymium*). In this case, all unambiguous C insertional editing sites in *Physarum* and *Didymium* for all 24 genes are counted.

Figure 5 illustrates the relationship between codon bias and the overall conservation for the 16 known genes (already described above), 16 known genes + 8 genes with editing sites in *Physarum* and 16 known genes + 8 genes with editing sites in *Physarum* and *Didymium* with 2 and 4 groups, respectively. As can be seen from this figure, the strength of codon bias of the 24 genes (including the known genes and predicted genes) is not as strong as in the 16 known genes. However, given the reduced error bars the dependence of codon bias on selection pressure remains statistically significant. We have thus shown that more conserved genes have more significant codon bias in all unambiguous C insertional editing sites in *Physarum* and *Didymium* as suggested by the previous theory [31].

## Methods

### Sequences

The mitochondrial genomes of two related organisms with insertional RNA editing, *Physarum polycephalum* and *Didymium iridis* were studied. Sixteen genes and their mRNA sequences from the two organisms were included in this study: atp1, apt6, atp8, atp9, cox1, cox2, cytb, nad4L, nad6, nad7, rpL2, rpL16, rpS3, rpS7, rpS12, and rpS19. All the sequences were downloaded from GenBank; see Table S4 for accession numbers. For several of our studies the sixteen genes were divided into groups according to their overall sequence conservation at the second codon position between *Physarum* and *Didymium*, which was obtained by aligning the mRNA sequences of each gene between the two organisms. Table S1 indicates for each gene which group it was assigned to.

### Scoring of prediction accuracy

The predicted editing sites were scored as either correct, one away, two away, or three or more sites away from the actual editing sites by comparison with the known mRNA sequences. We only scored C insertion sites, i.e., we ignored predicted insertion sites in close vicinity of thymine, adenine, guanine, or dinucleotide insertion sites in the known mRNA sequences. Also recorded was the number of editing sites included in each prediction due to occasionally missed editing sites at the beginning or at the end of a gene. Omissions of editing sites at either end of a gene was caused by not having a significant number of bases either before or after the input basis sequence. Therefore, the missed editing sites in these instances were due to the lack of information input into the computational method which results in poor conservation of the protein sequence of the gene. Thus, missed editing sites at the beginning and end of a gene sequence were not scored. While these types of predictions were not scored, occasionally an editing site would be missed or added by the prediction in the interior of a gene. Missed or added interior editing sites most often occurred in threes which preserves the reading frame and is most likely to conserve the protein sequence; interior missed or added editing sites were scored as three or more sites from the actual site.

### Determination of sequence conservation

For each gene, four sequences (*Physarum*-DNA, *Physarum*-mRNA, *Didymium*-DNA, and *Didymium*-mRNA) were aligned in Clustal X [33]. From these alignments, the C insertional editing sites that are unambiguous (i.e., at least in one of the two organisms the neighboring nucleotides are not Cs) and shared between *Physarum* and *Didymium* were identified. The flanking sequences within the window of 9 positions upstream and downstream of each of these editing sites in both organisms were investigated. Then these flanking sequences were turned into patterns of “0” and “1” where “1” means that the two organisms have the same base at the same relative positive and “0” means that they do not. The shared and unambiguous editing sites were separated by codon position.

### Background conservation

Comparing the mRNA sequences of the two organisms, we obtained the overall conservation information for the first, second and third codon position by counting the “1”s in each codon position across the whole genes. This yielded the background frequencies or the expected frequencies of “1”s at the first, second, and third codon position.

### Statistical significance of deviations from background

In order to see whether variations from background were statistically significant, the probabilities for observing increased (or decreased) sequence conservation in the vicinity of the shared insertional editing sites were calculated. These probabilities were calculated based on the binomial distribution: The background frequency or the expected frequency of “1”s at the codon position 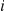 is 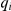. The total number of shared editing sites at the 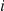
'th codon position is 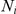. For a specific position in the vicinity of the shared editing site, we can easily identify its codon position 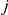 (see Table S5) and the actual number 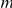 of “1”s in these 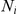 samples. Thus, the observed frequency of “1” at this specific position is 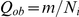. Therefore, the probability of the observed increased sequence conservation is:
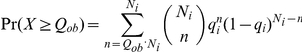
If the observed frequency of “1” is less than the “expected” frequency, the 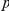-value was calculated analogously as the probability of observing the decreased sequence conservation.

### Codon bias and its error estimate

In order to determine the codon bias in insertional RNA editing, the number of third codon editing sites 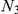 and the number of second codon editing sites 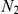 were counted. The codon bias was then quantified as their ratio 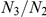. If we assume that the error for 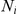 is just counting error given by the square root of 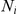 (i.e., 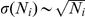), the statistical error of 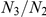 is
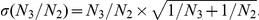



### “Expected conservation” for RNA editing site recognition

In order to know how much increase in conservation for recognition sites of editing events we should see in the mitochondrial genome of *Physarum* (62862 bp, NC_002508) if the region containing the editing site information is limited to the 18–19 nucleotides surrounding an editing site identified in Rhee *et al.* [18], we calculated the lowest conservation for a set of 19 nucleotides that allows specification of a site within the genome.

Since the effect of GC content in the *Physarum* mitochondrial genome is strong (the GC content is approximately 25% [34]), we considered the frequency that a set of 19 nucleotides occurs in a random DNA sequence with the same length (62862 bp) as well as the same GC content as the mitochondrial genome of *Physarum*. In such a sequence, the probability of two nucleotides being equal by chance is 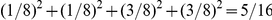. Therefore, the probability for two sets of 19 nucleotides in the sequence described above being the same is 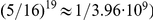 (i.e., the occurring rate for such a combination is 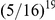), which is much lower than one in *Physarum*'s mitochondrial genome. Thus, we relax the constraints on a set of 19 nucleotides that will still specify the editing site (decrease the number of nucleotides that are fixed) as long as the frequency of the relaxed constraints is not (much) higher than 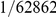. We found that the occurring rate of a motif in which only 9 of the 19 nucleotides are fixed (and the other nucleotides could occur randomly) is 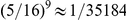, which is close to one per *Physarum* mitochondrial genome. We thus conclude that to uniquely specify a site by a 19 nucleotide motif, at least 9 of these nucleotides have to be fixed while the others can be variable.

We do not know which 9 (or more) of the 19 nucleotides are fixed for a given editing site, but we can calculate the average conservation generated by these fixed nucleotides. This average conservation for a set of 19 nucleotides is calculated as following: The conservation for each of the 9 fixed nucleotides is 100% while it is at least 63.3% (the lowest conservation between *Physarum* and *Didymium* we obtained, see Table 1) for the 10 random nucleotides. Thus, the average conservation is at least 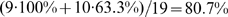.

## Supporting Information

Figure S1 An analysis of the predicted editing sites for the newly predicted *Didymium* genes as well as for the prediction of nad3 of which it is already known that it is unedited. The expected number of editing sites, the number of editing sites that are one or two away, and number of correct editing sites are shown based on the errors in the prediction results of the genes with known editing sites. The errors were calculated separately for the more conserved and the less conserved genes. The two genes labeled in red are less conserved genes.
(PDF)

Figure S2 Comparison of observed and expected conservation for the 8 less conserved genes for editing sites at the (a) first and (b) third codon position.
(PDF)

Figure S3

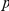
-values for the differences between the observed and the background conservation for shared editing sites in the 8 less conserved genes at the (a) first and (b) third codon position. The threshold for statistical significance (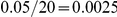 as the 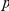-value cut off) is not indicated in the figure since it would be beyond the top edge of the graph.(PDF)

Table S1 Conservation at the second position and group division for the 16 known genes. The group division depends on the conservation at the second codon position. Genes with a background frequency at the 2nd codon position less of than 85.0% are assigned to the “less conserved” group while the other genes are assigned to the “more conserved” group. For the codon bias analysis a more fine grained division into four groups is also used.
(PDF)

Table S2 Predicted edited mRNA sequences of eight genes which were computationally identified in the mitochondrial genome of *Didymium* (including nad3 of which it is known that it is unedited). The upper case C's are the predicted insertional editing sites.
(PDF)

Table S3 Accession numbers for the eight *Physarum* genes which were also identified in *Didymium*.
(PDF)

Table S4 Accession numbers for the 16 mitochondrial protein coding genes with known editing sites in *Physarum* and *Didymium*.
(PDF)

Table S5 Relationships between codon positions of editing sites and the vicinity of editing sites. The numbers 1, 2, and 3 represent the codon position for corresponding site.
(PDF)
