# Supplementary material for: Comparison of Insertional RNA Editing in Myxomycetes
Source: PLoS Comput Biol. 2012 Feb 23;8(2):e1002400. doi: 10.1371/journal.pcbi.1002400 (PMC3285571; doi:10.1371/journal.pcbi.1002400)
Supplement: Figure S1 — An analysis of the predicted editing sites for the newly predicted Didymium genes as well as for the prediction of nad3 of which it is already known that it is unedited. The expected number of editing sites, the number of editing sites that are one or two away, and number of correct editing sites are shown based on the errors in the prediction results of the genes with known editing sites. The errors were calculated separately for the more conserved and the less conserved genes. The two genes labeled in red are less conserved genes. (PDF) [file pcbi.1002400.s001.pdf]

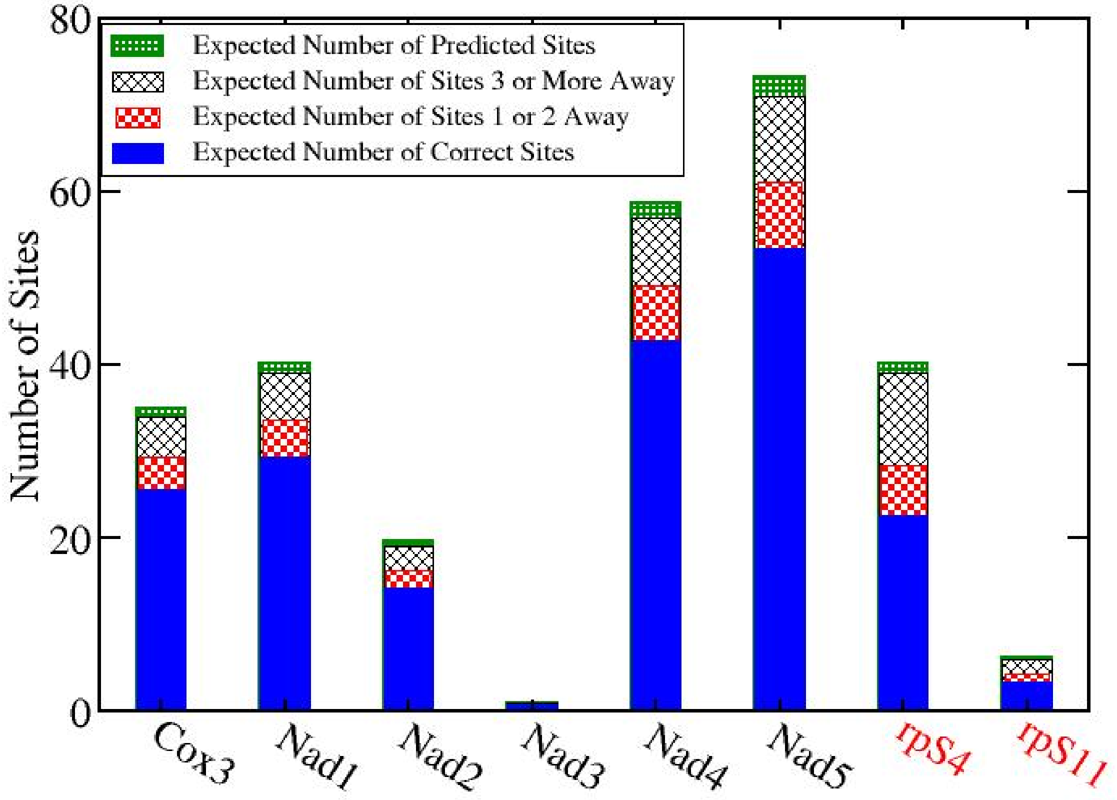

Figure S1 An analysis of the predicted editing sites for the newly predicted *Didymium* genes as well as for the prediction of nad3 of which it is already known that it is unedited. The expected number of editing sites, the number of editing sites that are one or two away, and number of correct editing sites are shown based on the errors in the prediction results of the genes with known editing sites. The errors were calculated separately for the more conserved and the less conserved genes. The two genes labeled in red are less conserved genes.
