# Supplementary material for: Comparison of Insertional RNA Editing in Myxomycetes
Source: PLoS Comput Biol. 2012 Feb 23;8(2):e1002400. doi: 10.1371/journal.pcbi.1002400 (PMC3285571; doi:10.1371/journal.pcbi.1002400)
Supplement: Figure S2 — Comparison of observed and expected conservation for the 8 less conserved genes for editing sites at the (a) first and (b) third codon position. (PDF) [file pcbi.1002400.s002.pdf]

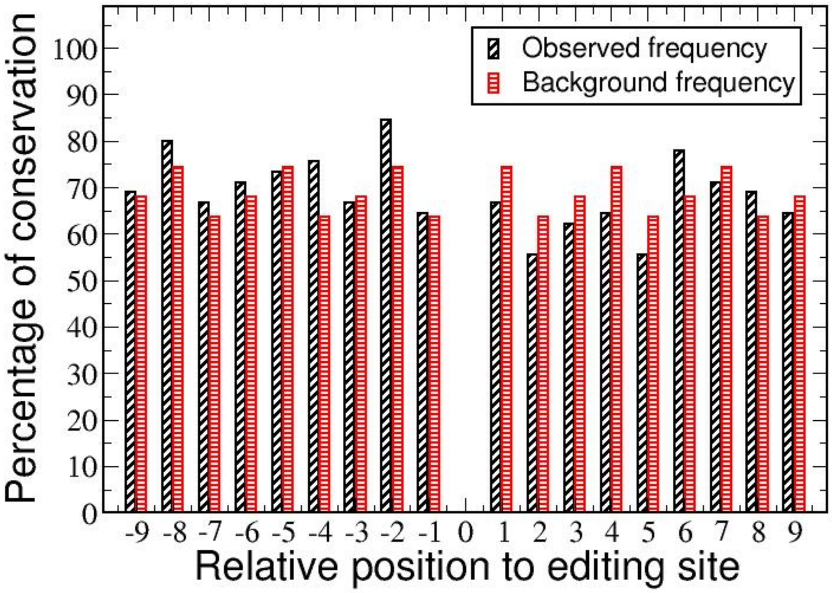

(a)

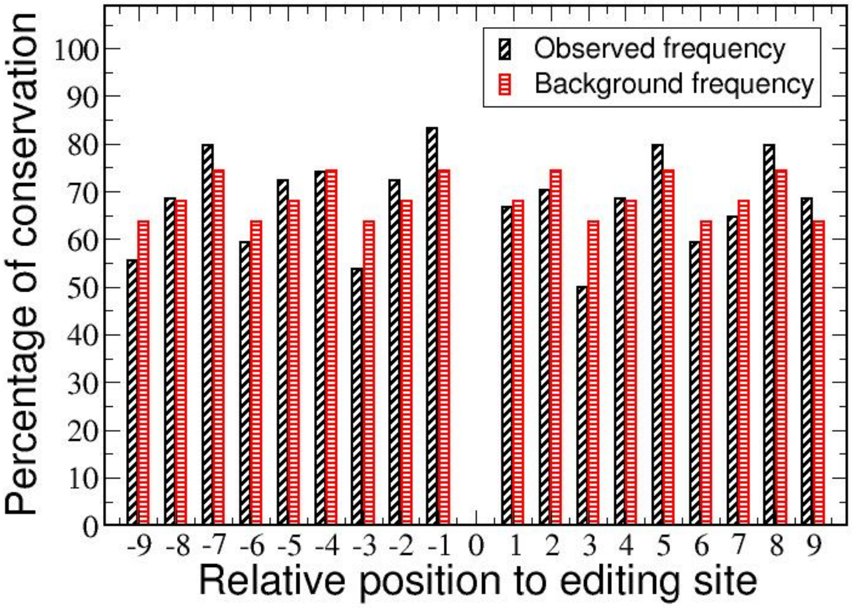

(b)

Figure S2 Comparison of observed and expected conservation for the 8 less conserved genes for editing sites at the (a) first and (b) third codon position.
