# Supplementary material for: Comparison of Insertional RNA Editing in Myxomycetes
Source: PLoS Comput Biol. 2012 Feb 23;8(2):e1002400. doi: 10.1371/journal.pcbi.1002400 (PMC3285571; doi:10.1371/journal.pcbi.1002400)
Supplement: Figure S3 — -values for the differences between the observed and the background conservation for shared editing sites in the 8 less conserved genes at the (a) first and (b) third codon position. The threshold for statistical significance ( as the -value cut off) is not indicated in the figure since it would be beyond the top edge of the graph. (PDF) [file pcbi.1002400.s003.pdf]

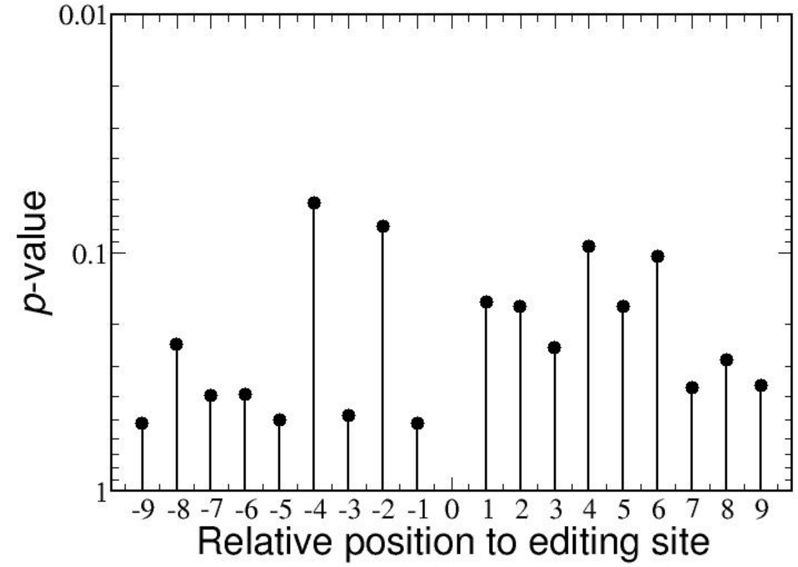

(a)

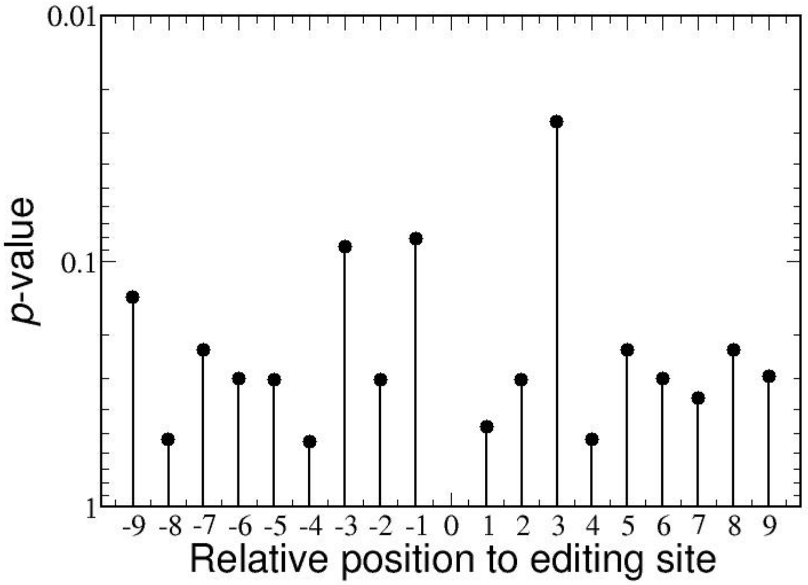

(b)

Figure S3 *p*-values for the differences between the observed and the background conservation for shared editing sites in the 8 less conserved genes at the (a) first and (b) third codon position. The threshold for statistical significance (0.05/20=0.0025 as the *p*-value cut off) is not indicated in the figure since it would be beyond the top edge of the graph.
