# Supplementary material for: Comparison of Insertional RNA Editing in Myxomycetes
Source: PLoS Comput Biol. 2012 Feb 23;8(2):e1002400. doi: 10.1371/journal.pcbi.1002400 (PMC3285571; doi:10.1371/journal.pcbi.1002400)
Supplement: Table S1 — Conservation at the second position and group division for the 16 known genes. The group division depends on the conservation at the second codon position. Genes with a background frequency at the 2nd codon position less of than 85.0% are assigned to the “less conserved” group while the other genes are assigned to the “more conserved” group. For the codon bias analysis a more fine grained division into four groups is also used. (PDF) [file pcbi.1002400.s004.pdf]

Gene	Conservation at the second codon position (%)	Major groups (“All” or “More conserved” or “Less conserved”)	Four groups for codon bias 1: 90%-100% 2: 80%-90% 3: 70%-80% 4: 60%-70%	Two groups for codon bias 1: 80%-100% 2: 60%-80%
atp1	86.0	More conserved	2	1
atp6	86.1	More conserved	2	1
atp8	73.4	Less conserved*	3	2
atp9	81.8	Less conserved	2	1
cox1	92.7	More conserved	1	1
cox2	97.0	More conserved	1	1
cytb	96.2	More conserved	1	1
nad4L	91.7	More conserved	1	1
nad6	86.3	More conserved	2	1
nad7	91.7	More conserved	1	1
rpL2	77.3	Less conserved	3	2
rpL16	76.9	Less conserved	3	2
rpS3	71.9	Less conserved	3	2
rpS7	60.9	Less conserved	4	2
rpS12	82.8	Less conserved	2	1
rpS19	78.0	Less conserved	3	2

Table S1 Conservation at the second position and group division for the 16 known genes. The group division depends on the conservation at the second codon position. Genes with a background frequency at the 2nd codon position less of than 85.0% are assigned to the “less conserved” group while the other genes are assigned to the “more conserved” group. For the codon bias analysis a more fine grained division into four groups is also used.

* Since we could not create a *Physarum* based PSSM for atp8, we excluded it in the RNA editing site prediction.
