# Supplementary material for: Comparison of Insertional RNA Editing in Myxomycetes
Source: PLoS Comput Biol. 2012 Feb 23;8(2):e1002400. doi: 10.1371/journal.pcbi.1002400 (PMC3285571; doi:10.1371/journal.pcbi.1002400)
Supplement: Table S2 — Predicted edited mRNA sequences of eight genes which were computationally identified in the mitochondrial genome of Didymium (including nad3 of which it is known that it is unedited). The upper case C's are the predicted insertional editing sites. (PDF) [file pcbi.1002400.s005.pdf]

>predicted cox3 gene in Didymium
atgtCtcataacaaacatCcatttcatttagtCaatccatctccatggcctatatCagtatctgcagctcttttaggattaactatCggtggtgtaatgt
attttcattCctttaataaaggtatCtatttagtaataatatcttttatCttagtagcCattattagtggtttttggtggcgtgatCttatacgcgaagg
tacCtatttacgtaatcacactttagaagtCttggctggtttacgtCtaggttttatgttatttattgtttcagaagcCatgcttttcttttctttttc
tgggcCtattttcatagtagtCtaagccctaatatagaaatCggtagtcaatggcctccttatgctttagaagtCattggtcttaccttacctatagtta
atacCattatactcttgacttcaggCgctactattactgtggctcatCttgcaattttaagaggcaaaaaacaaactgcaatCgaagctttaattgtaac
CttattattagctttagtatttacCagtattcaagcttatgaatatCgccatgcacCcttttctatttctgatggtatttatggttctgtCttttatatg
ttaacaggttttcatggtattcatgttatcatCggtactatctttatCttagttcaatttatacgtttaacCaaggatcaCatttttcaaaaaagtcatt
taggttttgaagcCttcgcttggtactggcattttgtagatgttgtttggttacttttatttttaatCgtttatgcttatggcCggtaa

>predicted nad1 gene in Didymium
atgtatattttaatCactattataaaattttttaataattatCaaaccaatttttattaacagtagctactttaactttatttgaacgaaaagtCatggccg
ctattCaaattcgtcgtggtcctaatgttataggCtttggtttgttacaacCccttgctgatggtttaaaattattgattaaagaaCttattataccatt
aaaaggtaataaatatCttttcttattttcaccagttctctttctCagtttaagttttgcCaagtggtctgtaattCcttttgaacaacattatttagcC
tctccgaatcttgtCatattagtatttttagcattCtcctctttatctgtttatggaatCttattgggtggttgggcttCtaattctcgttatgcCtttt
taggtgcattaagatCagcttcacaaatgatttcttatgaattagtCttaagtttattaatattattagtCtgtattcttgcacaatCctttaattttgt
agatatcgtatCagcacaatctacatCttggtttttttatacCtttaatgcCcttttgtttaatctatatgattgcaatattagCtgaaactaatagaact
ccttttgatCttccagaagccgaagCtgaacttgttgctgggtatagtgtCgaatattctagtggtCcctttgcttttttacttcatcgcagaataCtgta
atcttatCatttggtcgaataCtagtgttattctttttttttggtggatggtttCcttcttcattatcaatgatatacCctttttatattatatttgcctt
gaaatCtttagttatactattattattttgttggatacgcgcggcCttaccgcgttaccgttgggaccagttacttactttggcatggcgtacCtattta
cctgtagtcattgttagtgtttttttttgtCtactgtaatttttgtttttgatttatctatttttaa

>predicted nad2 gene in Didymium
atgcCttttatCttttatttatttagttataatatCttaattactatagcatttatccctatCttatttttaagaagatCagattattcttctgcttttta
ccaatCtacgtgatttaatCattctaaaaaaatctaacCccgttattgcatgtttttatgcttgtatggCtataagcttgagtggagttccgcCtttact
tggattttttttctaaactttttatCttaatttctgctatatCtttttctgcttatttactgtCggtttattacttttatttttctattttaagtgccttt
tactatttacgtCtagtaaaaatttttatatttttCcttttatttaaaatatgcctctttaatCtctataccattattcctgcCattattttttagtgttt
tttctgctatCaatatcttctttatCgtctatcctatgtattttattattctattgaatatactttaa

>predicted nad3 gene in Didymium
atgaatattttcttatcagaatatcgtaacatatttatatttatattatgctcatttgtattatctatagtgttattagcagcagtttatatattaagtt
tcacatcaaaagttgatttagagaaatcttctgcatacgaatgtggatttcaaccttttttcagaaactagttatccttttgaagtacaatttgctgttat
tgccataatgttcttattatttgatatcgaagttttatatcttttcccacttataagttctttatatacgttattatcgttagatgtaataataatattt
ggtttctacattatattattgatcggtttacttttttgaaatcagtcgtaaggtCtttagattta

>predicted nad4 gene in Didymium
atgaCtttacttttaaatagtttaatCattttatttattataattttattacttatacCtgctgaaagatCctttatggttcataatttttCcctaatag
gtgcttttcttatttttattgtCtctttagtattattatatatacCtatgtctttatctatCtttacCaataacaggttttttgttttattatcttttCc
ttcttttgttattaattaCtcttatatggtcgataatatatCtgtaatttatattatattatctacCttgcttggcattatagtagtCctggttacaaga
agtatagaatatCgtttaaaagaaaagtatatCatattatttttaattttaCtattattaataaactgttttacgactttggaaatCttatttttttata
tttttttttgaagcCttattaattcctgtCtttgcgcttattggtatttggggttCacaacaagaaaaaaatCtttgctgctaatCaattttttctatatac
attattCggttcttttttttatgttaacCggtattgtatttgttatgatCattacaggaactacaaatattttaattttaaaaagttatgtattCgaagct
cacattgaaaaaattatatttctatttttttatgctttcaCtttctgtaaaagtgccttccataccactacatttatggcttcctaaagctCatgtagaag
ctccaacaacCggtagtgttcttttagcaggtatCcttttaaaattaggttcttaCggatttttacgtttttCagtatttttatttCctaatgcttcttt
ttaCtttttacctttaatattatCaatagctgttatatctatagtattagccCtcttttacagtCttacgccaaaatgatcttaaaagaattatagcCtac
tcttctatagcacatatgaatttttttactaggagcCatttttgtaaaagatcttgtagttatCacaggttcacttcttttacaaatagcacatggaCtct
cctcttctgcattattttttagctatCggtatgctttatgatCgatataaatctcgaaatatCtattattatcgtggtCtagttgtaataatgccttttta
ttgtttcttttcttttattttctttagcaaatttaggttttCctggaactataaattttgcCgcagaaatgatgatatttttttggtatatttaatact
acCcccttctatagcaatCttgactttaacaggtatCttcttatctgcCaattattccttttgtattgttaactcgtatCgcttttggtcctgctagtCtat
atgttagagcCtttcacgatttaacacgacgtgaattttatatCttagctcctttaggtttcttaataatattttttaggtttatttccgaatcttttaaC
ttcatattggactttttctttggttacttggttctaa

>predicted nad5 gene in Didymium
atgtCattattctttcctCttttagcttttataatcttattttttttttggaagatttattggacgacaagtCtgtttaattttttgctgttttaatgtCcc
acatttctttaataatCtgtatatattattttttatacaagtCtttttttcaaggaaatgtttaCgtcttttctttaggttcttggatatctgtaggattcct
cgaaataacctataagtttatcttagatcctttatcaataacCtttgctacacttatatcaacaattactttattaatCattatttattcttatgaCtat
ttacatgaagatcctaatCttgttaaattttttttcttatCttagttttttttttcttttttctatgtCttgccttgtgcttgcaggtaattacttCgtaatgt

ttttaggatgggaagctgtCggtttagcatcttatCttcttattaatttttggtCtacacgtaatcaagcCaatcaatctgcattaaaagcgattatatt
taatagaaCaggtgatgcggcatttataactgcaatggcttgtatattttatcttttccattCctttgattttgaagatatcgaattacttattCctcaa
ttctcacatgttgaaataacCatttttcacaatCcttttctgctatagaaCtattagcagcttttatgttCttagctgcCagtgctaaatctgcacaaC
tatttttacatccatggttaccggatgctatCgaaggacctactcccgtatcagccttgcttcattccgcgaccatggttactgcaggagtCtttttatt
attaagatCctctgtaattttttacaaatgccccaaatgtttctttaCtagttgcttgtataggtcttatCactgcaaatatttcttcattaactggtCta
ttacaatatgatataaaacgtatcatCgcattttctacatgtagcCaattaggttttatgatgtttgctgtCggtgttggtaatttttCctttgcattat
ttcatttagtaaatCatgctttttttaaagcattattatCcttgtgtgcaggatctgttattcatgcgactggtgaacaagacatacgtagaatgggtgC
tttatataaagCattacctataacttatgtagcaatgctCcttgcatcattatctttagttggttttCctttcttaagtggtttttatCagaaagatttc
ttattagaagctacttatagtctttttggtaagttCagttttgttatttattttatttctatatctactttatgtCagtctttttattcttttcgtttaa
ttttctttgtatttttcggtgatgtagtgtCatctaaaagaatattaaaaaCatgcagtgaaagttcttatttcttatatattCctttaattgtattgac
aatattatcaatCttttcaggttttttttcaaaagatCttatggttattttttcttCtCtttataacttttctacttctCctttacaagaaatacttttt
gatatggaCtttttaatgatttttttcaaagtCttacctacaatattttctttatCtggtatttatttagtatatagcaaatatctgtCtgtaaatactg
catttcgcttattctataaaaaatatCtattatttgtttatCtattttgtaacaaattctttgCtgatgcctttaatagtttttatatCttcttacctac
tgcaaCattttctttaaaaatCacttatgaacttattgatCaaggttttcttgaattttatggttctactggtatCtattctttattgaatCcctttct
aatcgtttagcttatgtCgaaactactacacttatCtatcgttcattgttatttgtaattttcgctaCtaggtctttttcttatagtttatttagttgga
atattcttatgattatgtttagtagtcttagcatttattttgttttaggtCaaaatttaagtaattataaataa

>predicted rpS4 gene in Didymium
atgatCaattatattcaaaagaCtaataaaaatCagtttactttaagatataaaaaaataCaagaaattaggtagtgatCtttggggtcgtatCgcttttt
taaataaaaaaaaatgaaataacaattatCatgagaaatcttgctgaaaagatattaaaagtCCgtattcgttatttaactaaaCggaaaaaaaaaacgcat
CaaaaaacgttttagacttcgcCgtaaatttacgtttttttcaataCactatcttagcaaaacCcaaaaagaaatgtagaaatCcgtatgacCtaaagatC
CctaagttCagattattacgaaaaaaaataaaattattttataatCtacgtttaacacaaaaaagcatCagaaaattagtCagatatcgtaaatttagac
gtagaatCtcaaaaaataatattCctgctttccttgaaacacgttttgatgtCatgttatatcgtttaaattttatCtcttcaatatatgaaggtcgtag
aattatccgtacaaaaaagctttCtgttttaggacCatctaataaaaaatCaaataataaatttttttcagcatCaacaatgagaaaatcttatCcacaa
attcctatattttCatttatactttaaattataagttagccttacttCgtaaaatctatttaaaaaacatCtattatgcttggaagttagtagcCtatc
ctccatcatacttataCgcctcttatagaacaatgataggtCttacggttaataacccaaaagtaaataaaatCagttatcctttttaCaaggttattagc
atattttatgggcgcagcttttatttttaaataa

>predicted rpS11 gene in Didymium
atgataaaaatCcttgttgaaaaactttcagaattagttaaatcaaaaatttcttctttttcattaaccaggccaaataaaataaaacccaagcaca
taaaatttagttttggtagtgtttttctgagatggcgtacacgattattatatttcaaaacagtaaaacaacaacatcgtgacgttattgaagatattga
aagtagtttttcttattatttacaatttgtaatttctaaaatagaacgtcgtCgagaaacCataaacCgtttttttagcaggtaatttttcccttgtagta
ccaatggcttatagattattttgttatactaaagtttttattacacatggttttaggaatacatttattacaatatttcgtggagataaaaaggaagatg
aaaaaaatcaatttaatatgttaccagttactaaatatagttgtgggagtatcggatatcaaggtcctaaaaaatctactactttcgcaagaaaagaagt
aataaaaattgcaggtaactttttgggtacatctatgacaacattattaactgtcgttttcacttctaaagtatctagatggaatcgaaaaacaataaga
aatctatgccctaacttaagctttgtCCgttaa

Table S2 Predicted edited mRNA sequences of eight genes which were computationally identified in the mitochondrial genome of *Didymium* (including nad3 of which it is known that it is unedited). The upper case C's are the predicted insertional editing sites.
