# Supplementary material for: Comparison of Insertional RNA Editing in Myxomycetes
Source: PLoS Comput Biol. 2012 Feb 23;8(2):e1002400. doi: 10.1371/journal.pcbi.1002400 (PMC3285571; doi:10.1371/journal.pcbi.1002400)
Supplement: Table S3 — Accession numbers for the eight Physarum genes which were also identified in Didymium. (PDF) [file pcbi.1002400.s006.pdf]

Gene	Accession number for *Physarum*-mRNA
cox3	AF084527
nad1	HQ849404
nad2	DQ092490
nad3	HQ849405
nad4	HQ849406
nad5	HQ849407
rpS4	HQ849426
rpS11	HQ849418

Table S3 Accession numbers for the eight *Physarum* genes which were also identified in *Didymium*.
