# Supplementary material for: Comparison of Insertional RNA Editing in Myxomycetes
Source: PLoS Comput Biol. 2012 Feb 23;8(2):e1002400. doi: 10.1371/journal.pcbi.1002400 (PMC3285571; doi:10.1371/journal.pcbi.1002400)
Supplement: Table S4 — Accession numbers for the 16 mitochondrial protein coding genes with known editing sites in Physarum and Didymium. (PDF) [file pcbi.1002400.s007.pdf]

Gene	Accession number for *Physarum*-DNA	Accession number for *Physarum*-mRNA	Accession number for *Didymium*-DNA	Accession number for *Didymium*-mRNA
atp1	NC_002508	M31718	GU182118	GU182119
atp6	NC_002508	FJ154098	GU182120	GU182121
atp8	NC_002508	DQ092488	GU182122	GU182123
atp9	NC_002508	S67221	GU182124	GU182125
cox1	NC_002508	L14769	GU182126	GU182127
cox2	NC_002508	DQ092489	GU182128	GU182129
cytb	NC_002508	AF079799	GU182130	GU182131
nad4L	NC_002508	DQ092491	GU182132	GU182133
nad6	NC_002508	DQ092492	GU182134	GU182135
nad7	NC_002508	AB039844	GU182136	GU182137
rpL2	NC_002508	HQ849416	GU260652	GU182153
rpL16	NC_002508	HQ849414	GU260654	GU182155
rpS3	NC_002508	HQ849425	GU260656	GU182157
rpS7	NC_002508	HQ849427	GU260658	GU182159
rpS12	NC_002508	HQ849419	GU260660	GU182161
rpS19	NC_002508	HQ849423	GU260662	GU182163

Table S4 Accession numbers for the 16 mitochondrial protein coding genes with known editing sites in *Physarum* and *Didymium*.
