# Supplementary material for: Comparison of Insertional RNA Editing in Myxomycetes
Source: PLoS Comput Biol. 2012 Feb 23;8(2):e1002400. doi: 10.1371/journal.pcbi.1002400 (PMC3285571; doi:10.1371/journal.pcbi.1002400)
Supplement: Table S5 — Relationships between codon positions of editing sites and the vicinity of editing sites. The numbers 1, 2, and 3 represent the codon position for corresponding site. (PDF) [file pcbi.1002400.s008.pdf]

Relative Position to editing site	Editing site as the **first** codon position	Editing site as the **second** codon position	Editing site as the **third** codon position
-9	1	2	3
-8	2	3	1
-7	3	1	2
-6	1	2	3
-5	2	3	1
-4	3	1	2
-3	1	2	3
-2	2	3	1
-1	3	1	2
**0**	**1**	**2**	**3**
1	2	3	1
2	3	1	2
3	1	2	3
4	2	3	1
5	3	1	2
6	1	2	3
7	2	3	1
8	3	1	2
9	1	2	3

Table S5 Relationships between codon positions of editing sites and the vicinity of editing sites. The numbers 1, 2, and 3 represent the codon position for corresponding site.
